# Vascular Ageing and Exercise: Focus on Cellular Reparative Processes

**DOI:** 10.1155/2016/3583956

**Published:** 2015-12-01

**Authors:** Mark D. Ross, Eva Malone, Geraint Florida-James

**Affiliations:** School of Life, Sport and Social Sciences, Edinburgh Napier University, Edinburgh EH11 4BN, UK

## Abstract

Ageing is associated with an increased risk of developing noncommunicable diseases (NCDs), such as diabetes and cardiovascular disease (CVD). The increased risk can be attributable to increased prolonged exposure to oxidative stress. Often, CVD is preceded by endothelial dysfunction, which carries with it a proatherothrombotic phenotype. Endothelial senescence and reduced production and release of nitric oxide (NO) are associated with “vascular ageing” and are often accompanied by a reduced ability for the body to repair vascular damage, termed “reendothelialization.” Exercise has been repeatedly shown to confer protection against CVD and diabetes risk and incidence. Regular exercise promotes endothelial function and can prevent endothelial senescence, often through a reduction in oxidative stress. Recently, endothelial precursors, endothelial progenitor cells (EPC), have been shown to repair damaged endothelium, and reduced circulating number and/or function of these cells is associated with ageing. Exercise can modulate both number and function of these cells to promote endothelial homeostasis. In this review we look at the effects of advancing age on the endothelium and these endothelial precursors and how exercise appears to offset this “vascular ageing” process.

## 1. The Endothelium in Health and Disease

The endothelium controls diffusion and transport of nutrients, gases, and other signalling molecules from the blood into the surrounding tissues and controls adhesion, rolling, and migration of leukocytes to sites of infection and tissue damage. The endothelium also controls blood flow distribution around the body through the release of vasoactive substances, including nitric oxide (NO) and prostacyclin (PGI_2_) [1]. Under normal conditions, NO is released from the endothelium, which diffuses to the vascular smooth muscle, causing the smooth muscle cells to relax, thus widening the diameter of the blood vessel, allowing more blood to flow distally to that vessel, a process termed endothelial function. NO is not only vasoactive to control vessel lumen diameter, but also antiatherogenic, inhibiting platelet and leukocyte adhesion to the endothelium [2].

Endothelial dysfunction often precedes CVD, and the ability of the endothelium to produce and release NO, measured as flow-mediated dilatation (FMD), can be predictive of future cardiovascular events [3] and mortality [4], potentially due to those with endothelial dysfunction being susceptible to atheroma formation and progression. Therefore the endothelium is a key player in maintenance of vascular health.

## 2. The Ageing Endothelium Role of Oxidative Stress

Advancing age is associated with endothelial dysfunction [5–12], increased susceptibility of endothelial cells to apoptosis [13, 14], and altered intracellular signalling [8]. These have been linked to NO bioavailability [15–17] and chronic exposure to oxidative stress [9, 18–20], which is an imbalance between the production of free radicals (oxidants) and opposing antioxidants, of which the greater production or presence of oxidants than antioxidants results in tissue damage and cellular dysfunction. Indeed the bioavailability of NO itself is a product of the rate of NO production and its scavenging by free radicals. Free radicals, such as superoxide anions (O_2_^∙−^), have been found in greater levels in aged vascular tissue of rats compared to their younger counterparts [21], as well as in other models of ageing [22, 23], and levels of reactive oxygen species (ROS) derived from NADPH oxidase account for attenuated endothelial-dependent vasodilation in aged mice [24]. Scavenging or inhibition of these radicals improves endothelium function [15, 21]. The increased production of O_2_^∙−^ and NO leads to the formation of peroxynitrite [15, 25] and the subsequent uncoupling of eNOS [15]. In contrast to this, Luttrell et al. [26] observed high eNOS content within aortic rings of old versus young rats despite reduced endothelial function, a potential compensatory mechanism to increase the drive for NO production.

Oxidative stress can also promote atherosclerosis through the oxidation of low-density lipoprotein (oxLDL), which can stimulate macrophages to migrate from the circulation into the vascular wall [27], progressing to the development of foam cells, which is a key process in the formation of an atherosclerotic lesion. OxLDL also exerts deleterious effects on the vascular smooth muscle cell wall, by stimulating inflammatory cytokine release (tumour-necrosis factor-*α*, TNF-*α*, and monocyte chemoattractant protein-1, MCP-1) [28]. In fact, circulating oxLDL has been shown to be a predictor of cardiovascular events in humans [29], confirming its association with vascular health.

In addition to increased production of oxidants, there may be a concomitant reduction or impairment in antioxidant defences with ageing resulting in prooxidants going unchecked causing damage to surrounding tissues and accelerating ageing. For example, plasma concentration of one such antioxidant enzyme, superoxide dismutase (SOD), which itself catalyzes the dismutation of O_2_^∙−^ into oxygen and hydrogen peroxide, declines with age. This reduction was however not found in cellular tissue [11, 30], which itself may suggest that ageing may be associated with increased production of ROS rather than an impairment of cellular antioxidant capacity. Yet one study has found impaired activities of various antioxidant enzymes in aged cardiovascular tissue in rats [31]. More studies are required to fully elucidate the effects of age in animals and humans on antioxidant capacity. Acute administration of antioxidants, provided by vitamin C and SOD mimetics, can improve NO bioavailability and endothelial function [9, 32, 33], therefore offering the potential to reduce tissue damage via dietary means. This is not within the scope of this review, but for a review of this see Brown and Hu [34].

Sirtuin 1 (SIRT1) is a protein involved in DNA repair, cell cycle regulation, and ageing [35]. It functions to catalyze the removal of acetyl groups attached to lysine residues of various molecules involved in cellular signalling [36]. It is expressed in endothelial cells and has been observed to play a key role in prevention of endothelial cell senescence [37–39] by modulating p53 expression [39] and P66Shc [37], both of which are involved in oxidative stress-induced senescence. SIRT1 expression and activity are reduced in aged endothelial cells [40], but by increasing the expressing of SIRT1, endothelial senescence can be prevented [38, 41]. It appears that SIRT1 also plays a protective role in preventing ROS production in endothelial cells, as activating SIRT1 pharmacologically prevents ROS-induced endothelial dysfunction [42]. In addition, inhibition of SIRT1 caused an increase in NADPH oxidase activity and the associated O_2_^∙−^ production [42], which can go on to inactivate NO [21]. SIRT1 is therefore an important regulator of vascular endothelial ageing.

In addition to loss of NO bioavailability with ageing, there has been documented evidence of loss of prostacyclin-mediated dilatation of vasculature in humans [43] potentially as a result of reduced prostacyclin production [44, 45]. However, under NO inhibition, the dilatory response to prostacyclin in young and old subjects was similar [43], implicating loss of the NO pathway as the primary mechanism behind ageing-induced vascular dysfunction.

## 3. Exercise and the Endothelium

**Figure 1:**
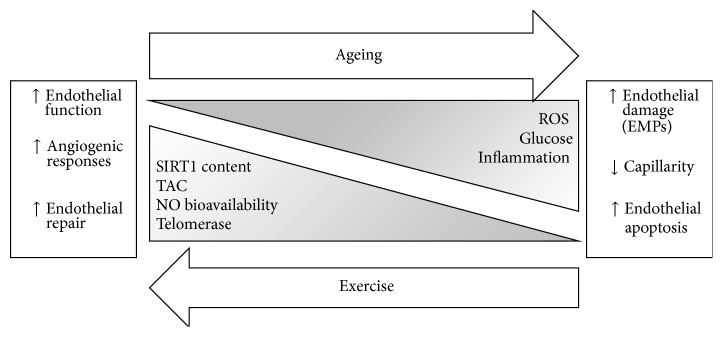
The effect of age and exercise on the endothelium. SIRT1: Sirtuin 1, TAC: total antioxidant capacity, NO: nitric oxide, ROS: reactive oxygen species, and EMP: endothelial microparticles.

Physical activity and regular exercise training has been shown to play a significant role in the prevention of CVD, in addition to reducing the risk of mortality [46–58]. The reverse can be seen with physical inactivity and sedentary lifestyle associated with increased risk of NCD and mortality [59–68]. Physical activity and regular exercise training is antiatherogenic [69] and reduces oxidative stress through upregulation of antioxidants, such as SOD [70, 71]. Exercise has been shown to increase mitochondrial manganese SOD and cytosolic Cu/Zn SOD isoforms [72] which may contribute to a reduction in oxidative stress in the endothelium. There is a plethora of studies showing the beneficial effects of exercise on FMD, indicating improved endothelial function [5, 6, 12, 73–81]. On the other hand, sedentary interventions (reducing step counts and increasing sitting time or bed rest studies) result in the opposite effect on FMD [82–84]. Exercise and periodic increases in physical activity result in increases in cardiac output and greater blood flow through the vasculature. The increase in flow across the endothelium generates a shear stress stimulus, which is the shearing effect of circulating cells across the endothelium. Greater levels of laminar shear stress, as observed during exercise, result in an increase in NO production and release by the endothelium to widen the vessel diameter. Birk et al. [85] investigated the role of shear stress on the vascular adaptation to exercise. They observed that, in individuals who exercised for 8 weeks, brachial artery dilatory response was greater in the arm that was unrestricted to blood flow, whereas, in the arm that was restricted to blood flow via an arm inflatable cuff, there was no significant change in endothelial-dependent dilatation. Therefore shear stress is a key stimulus for vascular adaptation during an exercise training programme. The improved endothelial function can be attributable to increased endothelial NO synthase (eNOS) protein levels within the endothelium as evidenced from mice models [19, 72] and/or reduced oxidative stress [19, 72, 76, 86]. The reduced oxidative stress can help prevent the uncoupling of NO, therefore increasing NO bioavailability. One study has however shown no changes in eNOS protein content as a result of exercise training [86]. The effects of ageing and exercise on the endothelium are summarised in Figure 1.

## 4. Endothelial Precursors: New Cellular Markers of Endothelial Regeneration

The endothelium is reported to have a turnover rate of between 47 and 23,000 days using continuous labelling techniques [87]. However, endothelial cell turnover may be higher in areas of bifurcations [88], potentially as a result of disturbed flow [89], and increases in oxidative stress [90]. It was thought that normal endothelial cell turnover was maintained by proliferation of resident endothelial cells; however, recently, the contributions of stem cell-like cells have been described, as also seen with skin [91] and skeletal muscle [92]. Endothelial precursors or endothelial progenitor cells (EPCs) were discovered in 1997 by Asahara et al. [93]. Researchers observed that isolated CD34^+^ cells from human peripheral blood formed tube-like structures on fibronectin-coated plates* in vitro*. These cells, after a period of 7 days in culture, began to express endothelial lineage markers such as VEGFR2, PECAM-1, and E-selectin and stained positively for eNOS. These CD34^+^ cells also secreted NO under stimulation by vascular endothelial growth factor (VEGF) or acetylcholine, a key characteristic of mature endothelial cells. These EPCs have been consistently shown to repair damaged endothelium in animal [93–95] and human studies [96–98]; however, these endothelial precursors are rare events in human peripheral blood, accounting for only between 0.0001 and 0.01% of all mononuclear cells [99], with this level varying depending on age and health status [100]. Tissue damage may stimulate a mobilisation of CD34^+^ progenitors, which may increase the circulating number of these cells by up to 500% (0.01% to 0.50% of all mononuclear cells) [101]. EPCs may make up a substantially smaller number in circulating pool and may only make up 10% of CD34^+^ progenitors [102].

There is some debate on the origin of these endothelial precursors. There is clear evidence showing that they are likely derived from the bone marrow [94, 95]. Some researchers have suggested that EPCs are resident in the vessel wall, with adventitia-resident CD34^+^ progenitors able to promote vessel formation* in vitro* [111] and* in vivo* [96]. However, Passman et al. [112] failed to observe endothelial differentiation of adventitial progenitors, with these cells instead taking a more vascular smooth muscle cell phenotype. It may be that the vascular growth and reparative process involves both circulating cells derived from the bone marrow and vascular-resident cells, promoting the proliferation of endothelial cells through paracrine means via the secretion of VEGF and also through differentiation into mature endothelial cell phenotype.

There appears to be 2 subsets of EPCs, each subset playing a different role in vascular regeneration and repair. These have been termed “early” and “late” outgrowth cells and named so because of their appearance in culture. The so-called “early” EPCs appear early in culture and die after 4 weeks. These cells secrete relatively large amounts of proangiogenic cytokines and growth factors such as VEGF and interleukin-8 (IL-8), whereas the “late” EPCs appear late in culture, live up to 12 weeks, produce more NO than “early” EPCs, and formed capillary structures to a greater extent than “early” EPCs [113]. It can be concluded that “late” EPCs have greater ability to differentiate into endothelial cells, whereas “early” EPCs have greater potential to promote vascular repair in a paracrine manner.

## 5. Endothelial Progenitor Cells and Vascular Disorders

Many studies have found that those with vascular-related disorders have reduced circulating number and/or impaired function of EPCs compared to healthy age-matched controls [114–138]. Numbers are also associated with endothelial function [116, 131], implicating the role of EPCs in maintaining endothelial health. Circulating “late” EPCs have also been shown to be predictive of mortality incidence, with those with higher numbers having a lower mortality rate than those with low circulating levels [139].

Paradoxically, Pelliccia et al. [140] found that those with high levels of CD34^+^CD45^−^VEGFR2^+^ cells were more likely to suffer a cardiovascular event within 5 years of follow-up after undergoing percutaneous coronary intervention. These findings may be attributable to the potential role of EPCs in the progression of atherosclerosis [126]. EPCs may contribute to atherosclerotic development through secretion of proinflammatory factors such as plasminogen activator inhibitor 1 (PAI-1) and monocyte chemoattractant protein-1 (MCP-1) [141]. Both are involved in atherosclerosis, with PAI-1 expressed within plaques, with more expression in increasingly progressed plaques [142], and with MCP-1 involved in the adhesion of monocytes to the vascular wall [143]. EPCs' secreting these proinflammatory mediators of atherosclerosis is a surprising function and paves the way for the potential role of EPCs in atherosclerosis development and progression. However there are some reports linking EPCs to the prevention of atherosclerosis, either by inverse relationships between number/function and atherosclerotic lesion development [119] or by infusion of these cells potentially causing reduced plaque burden or attenuation of plaque progression [144, 145]. Additional research is needed to fully elucidate the role in atherosclerosis development or prevention.

In the studies that show reduced circulating number of EPCs with vascular-related disorders, this reduction could be as a direct result of bone marrow depletion of these cells, due to an increased requirement for vascular repair. Mobilization of these cells by VEGF in mice has been found to reduce the number of both haematopoietic and mesenchymal stem/progenitor cells within the bone marrow after only 5 days [146]. In addition critical limb ischemia patients display reduced circulating EPCs as well as a reduced number of bone marrow resident CD34^+^ cells compared to healthy controls [132].

The observed reduction in circulating progenitor number in those with vascular disease may also be attributable to an impaired mobilization process. Matrix metalloproteinase-9 (MMP-9) activity in the bone marrow of critical ischemia patients is reduced accompanying a reduced circulating and bone marrow resident CD34^+^ cell number [132]. MMP-9 is involved in the mobilization of progenitors from the bone marrow [147–149], believed to be specifically involved in cleaving stromal-derived factor-1 (SDF-1, ligand for C-X-C Chemokine Receptor 4; CXCR4) allowing CXCR4^+^ progenitor cells free to leave the bone marrow [150]. Diabetics, both type I and type II, also appear to have reduced circulating EPC numbers compared to healthy controls [120, 131, 151–156], in part due to impaired mobilization. Type II diabetics present with a reduction in capillarity within the bone marrow, which was associated with the duration of the diabetes, as well as fasting glucose levels [155], with a possible consequence of inadequate nutrient delivery for progenitor or stem cell production within the bone marrow. This could implicate impaired progenitor cell maintenance in addition to impaired mobilization.

## 6. Ageing and Endothelial Progenitor Cells

As discussed, ageing is associated with endothelial dysfunction, as well as impaired angiogenesis [157–161]. These effects could be associated with a reduction in EPC numbers or impaired function of these vasculogenic cells. Age does in fact result in reduced circulating EPCs [104, 106] and impaired function as displayed as reduced migration and proliferation [97, 98, 103, 105, 106, 109, 110] (Table 1). In two studies, migration and proliferation of EPCs were independent predictors of endothelial function in both young and old individuals [103, 104].

Xia et al. [97, 98] used* in vivo* mouse models to investigate the effect of age and the ability of human “early” EPCs to repair damaged endothelium. The authors induced carotid artery injury in mice and found that the ability of the mice to repair the endothelium was age-dependent. The mice that received the “young” EPCs (EPCs isolated from young individuals) displayed a greater ability to repair the endothelium in comparison to those that received the “old” EPCs (EPCs isolated from old individuals). Based on the morphological appearance of these cells being “early” EPCs, it is likely that these cells promoted reendothelialization mainly via paracrine means [113]. This* in vivo* model was accompanied by* in vitro* age-related impairments in EPC migration and adhesion of these cells. The authors reported that, under stimulation by SDF-1, these “old” cells failed to phosphorylate Janus Kinase-2 (JAK-2) to the same extent as “young” EPCs, despite similar CXCR4 cell surface expression between the two age groups, implicating a disrupted intracellular signalling mechanism as the reason by which these cells become dysfunctional, rather than cell surface protein expression changes.

EPCs from old individuals may also display impaired paracrine action, as found by Kushner et al. [108], who observed reduced release of granulocyte colony-stimulating factor (G-CSF) after stimulation by the stimulant phytohemagglutinin (PHA). However, the stimulated release of IL-8, another proangiogenic cytokine, was not different between young and old individuals. Therefore, paracrine action of these cells and their ability to stimulate endothelial repair by signalling endothelial cells to proliferate may be hindered with age.

The same group measured telomere length in EPCs isolated from peripheral blood mononuclear cells (PBMNC) in another study. Telomeres are repetitive DNA sequences (TTAGGG) at the end of chromosomes, and they act to protect DNA from damage. Replication of cells causes the length of telomeres to shorten, and therefore telomere length has often been used as a biomarker for cellular/biological age [107, 162, 163]. Repeated rounds of division and replication may cause cells to become senescent (cells are unable to replicate further). Telomere length, as measured using genomic DNA preparation and Southern hybridization techniques in EPCs isolated from PBMNC from old compared to young individuals, was shorter; however, they were not different between young and middle-aged individuals [107]. The participants in this study were reported to be healthy men, with no history of CVD or diabetes, further strengthening the belief that these cells are affected not only by disease, but also by ageing. Recent evidence suggests that telomeres can be deleteriously impacted upon by ROS. In an ageing model using* nfkb1* knockout mice, fibroblasts that show accelerated ageing also display reduction in telomere length, yet this effect was attenuated by antioxidant treatment of the mice [164]. However, data is lacking with respect to circulating progenitor cells.

Once again, oxidative stress may play a central role in the ageing effect on progenitor cell number and function. CD34^+^ progenitors in male and female octogenarians were inversely correlated with circulating levels of ROS, and those individuals who had died by the end of the follow-up (7 years) had significantly higher levels of ROS at baseline [165], highlighting the importance of reducing oxidative stress and related damage for longevity. Indeed circulating levels of ROS are greater in aged humans than young humans, and this is also accompanied by a decreased EPC content of SIRT1, which may allow ROS damage to continue unchecked. SIRT1 administration to EPCs* in vitro* rescues EPCs from H_2_O_2_-induced apoptosis [166], and SIRT1 deletion leads progenitor cells to exhibit an ageing phenotype as indicated by an increase in DNA damage and increased intracellular content of ROS [167]. These observations lead us to believe that the process of ageing, through the increased production of ROS, and reduced SIRT1 content of EPCs, could lead, partially, to the reduced number and function of these vascular regenerative cells, increasing risk for CVD in ageing individuals.

The evidence points to a deleterious effect of ageing on the ability of the body to stimulate endothelial repair, through depletion of EPC number, in both circulation and bone marrow, as well as impairment of function of these cells. Ageing is associated with increased risk of NCDs [168], and the effect of age on EPCs may be a causative factor. It is therefore of great importance to maintain EPC number and function throughout the lifespan in order to reduce risk of these NCDs. There is plenty of evidence to show that pharmaceutical interventions, such as statins [169–176], can help maintain EPC number and function; however this places a large financial burden on health services, thus addressing other lifestyle factors, such as diet, and exercise may be more cost effective.

## 7. Exercise and Endothelial Progenitor Cells

Regular exercise has been consistently shown to be beneficial for health. Exercise can improve cardiorespiratory fitness, lower blood pressure [177, 178], improve left ventricular function [133, 179], reduce chronic low-grade inflammation [180], improve tissue perfusion [181], reduce fasting blood glucose [182], and increase insulin sensitivity [183]. Taken together, there is overwhelming evidence that regular exercise or having a higher level of cardiorespiratory fitness can offer some protection against NCD incidence and mortality [47–58]. Recently, there has been a growth in interest in EPC biology and the impact of exercise on these cells.

Acute exercise has been repeatedly shown to mobilize EPCs into the circulation in addition to enhancing the* in vitro* and* in vivo* function of these cells for a period of up to 72 hours, depending on the intensity and duration of the bout of exercise investigated [134, 184–202], with few studies showing no changes [104, 203] or even reductions in progenitors after exercise [204]. The observed increases in circulating angiogenic progenitors are often seen alongside increases in circulating SDF-1 [187], VEGF [184, 191, 193, 194], G-CSF, MMP-9 [193], or increased NO production [202]. Acute maximal exercise bout has been shown to improve the function of EPCs, as measured by increased migratory capacity to VEGF and SDF-1* in vitro* [134] which is proposed to aid in the cells being able to migrate to ischaemic tissue to stimulate vessel growth. The improved function of these cells may be due to increases in CXCR4 cell surface expression, yet this has yet to be investigated in EPCs. Exercise-induced increases in circulating cortisol have been found to increase CXCR4 expression in T-lymphocytes [205] indicating that the circulating environment that these cells are exposed to as a result of exercise may affect cell surface receptor expression and subsequently function. Increased CXCR4 cell surface expression could also be stimulated by increases in shear stress [97] caused by increases in cardiac output seen with exercise. Further study is required to investigate the effect of acute exercise and the role CXCR4 plays in postexercise improvements in EPC migratory function. Age also appears to have an impact on the acute exercise response. EPC numbers increased in circulation in old individuals; however, this response was attenuated in comparison to a young population [200] suggesting an impaired mobilisation process.

**Figure 2:**
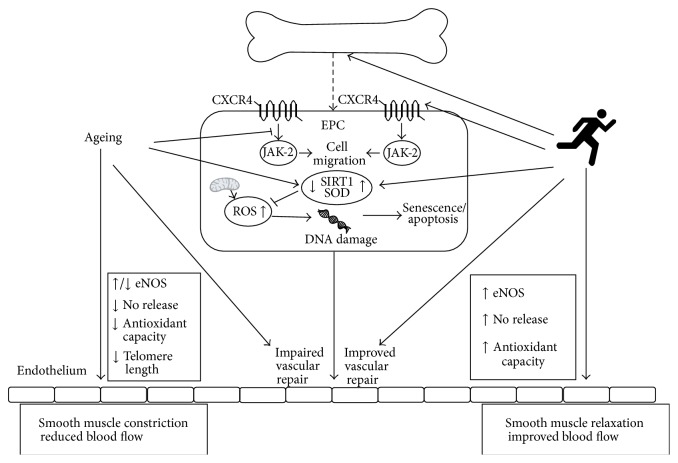
The effects of age and exercise on EPC-mediated vascular repair and endothelial function. Ageing causes the reduced signaling between CXCR4 and Janus Kinase-2 (JAK-2), as well as being associated with a reduced antioxidant capacity. Exercise mobilizes EPCs from bone marrow and rescues the signaling between CXCR4 and JAK-2, as well as stimulating production of antioxidants Sirtuin 1 (SIRT1) and superoxide dismutase (SOD). EPC-mediated repair of endothelium leads to improved endothelial function.

Regular exercise training also results in increases in resting EPC numbers [98, 105, 110, 206–220], potentially contributing to the observed improvement in endothelial function with exercise [219]. However, some studies have found no changes in circulating number but did find improvements in* in vivo* endothelial-repair ability [98],* in vitro* endothelial colony forming unit ability [215], or* in vitro* NO production [218]. Xia et al. [98] investigated the effect of regular exercise training on EPC-mediated endothelial repair using a murine model of carotid artery injury. Before and after exercise training (30 minutes per day, 3 days per week, and 12 weeks of aerobic exercise) human “early” EPCs were isolated and cultured. These cells were then injected into left carotid artery of athymic nude mice after carotid injury. Endothelial regeneration was greater in the mice injected with EPCs from young subjects compared to those injected with EPCs from older subjects. Endothelial regenerative ability of these cells was improved in the older men after the 12-week training period. The improvement in EPC* in vivo* function as a result of the exercise training period in humans was associated with improvements in intracellular signalling, with increased signalling between CXCR4 and its downstream target, Janus Kinase-2 (JAK-2) [98], a potential mechanism for the improved migratory capacity of these cells after training interventions [134]. Other functional improvements seen with exercise training include improved migration to VEGF [105, 217, 220] and SDF-1 [220], adhesion to human umbilical cord vein endothelial cells [98], and secretion of NO [218]. Importantly these improvements in EPC function and/or number have been found to be related to the improvement seen in endothelial function as a result of an exercise training program [219], potentially implicating these cells in the process of improving endothelial function with exercise. The effects of age and exercise on these progenitor cell subsets and their effect on the endothelium is summarised in Figure 2.

Other mechanisms behind improved number and function of these cells with exercise training are potentially linked to reduced oxidative stress which affect progenitor cell function [166] and lower fasting blood glucose, as hyperglycaemic conditions typically affect progenitor cell functions [152, 221].

Detraining and inactivity on the other hand play a role in reducing vascular regenerative capacity of these cells. Only 10-day detraining was sufficient to reduce CD34^+^ and CD34^+^VEGFR2^+^ progenitor cells, and the extent of decline in EPCs (CD34^+^VEGFR2^+^) was associated with the decline in endothelial function [222]. Additionally, these cells at baseline were associated with oxLDL plasma concentrations. The observed increase in EPC senescence potentially resulted from a reduction in total antioxidant capacity of the individual, which concomitantly decreased after the 10-day detraining. Data from this study suggest that oxidative stress and antioxidant capacity of the individual may be associated with physical activity and as a result may modulate EPC number and senescence and subsequent endothelial function and cardiovascular risk.

## 8. Summary

The process of ageing is often associated with increased morbidity and mortality. “Vascular ageing” represents the multitude of effects of ageing on the vascular tree including endothelial dysfunction, increased arterial stiffness, atherosclerotic plaque formation, and an impaired angiogenic response. Exercise training may offset this process of “vascular ageing” by maintaining or improving EPC number and function, which can then act to help maintain endothelial function through paracrine signalling to promote endothelial proliferation or by adhering to the vessel wall and differentiating into mature endothelial cells, with fully functional eNOS and high NO content. The reduction in oxidative stress as seen following exercise training programs may also promote EPC survival and prevent functional decline of these cells with age.

## Figures and Tables

**Table 1:** Effect of age on EPC number and function.

Reference	Subject population	EPC assay	Finding
Heiss et al., 2005 [103]	20 young (~25 yr) men 20 old (~61 yr) men	CD34^+^VEGFR2^+^, CD133^+^VEGFR2^+^ cells (FC) EPC CFU EPC migration	(i) No difference in EPC number between age groups (ii) Reduced EPC migration and proliferation in old versus young men

Thijssen et al., 2006 [104]	16 young (19–28 yr) men 8 old (67–76 yr) men	CD34^+^VEGFR2^+^ cells (FC)	EPC reduced in old versus young men

Hoetzer et al., 2007 [105]	10 young (22–35 yr) men 15 middle-aged (36–55 yr) men 21 old (56–74 yr) men	EPC CFU EPC migration	(i) Reduced proliferation in middle-aged and older versus young men (ii) Reduced migration in old versus middle-aged and young men

Thum et al., 2007 [106]	10 young (23–31 yr) men 16 middle-aged (50–69 yr) men 12 old (~74 yr) men.	CD133^+^VEGFR2^+^ cells (FC) EPC migration eNOS content of EPC	EPC numbers, migration, and eNOS content reduced in old versus young and middle-aged versus young men

Kushner et al., 2009 [107]	12 young (21–34 yr), 12 middle-aged (43–55 yr), and 16 old (57–68 yr) men	Telomere length of isolated EPCs	EPC telomere length significantly reduced in older versus middle-aged and young men

Kushner et al., 2010 [108]	17 young (21–34 yr) men 20 old (56–70 yr) men	EPC release of proangiogenic factors: G-CSF, VEGF, IL-8, and IL-17	EPC release of G-CSF impaired in old versus young men

Xia et al., 2012 [97, 98]	10 young (~27 yr) men 10 old (~68 yr) men 25 young (~26 yr) men 22 old (~68 yr) men	CD34^+^VEGFR2^+^ cells (FC) Mouse model of carotid injury and infusion of EPCs from young or old men. As above + EPC migration EPC adhesion assay	(i) EPC numbers reduced in old versus young men (ii) Reduced endothelial repair capacity in mouse model in old versus young men (iii) Reduced CXCR4:JAK-2 signalling in old versus young men (iv) EPC adhesion to endothelial monolayer impaired in old versus young men (v) Reduced EPC migration in old versus young men

Williamson et al., 2013 [109]	4 young (20–30 yr) individuals 4 old (50–70 yr) individuals	EPC CFU EPC migration	(i) No difference in proliferation between young and old individuals (ii) Reduced migration in old versus young individuals

Yang et al., 2013 [110]	20 young (21–33 yr) men 20 old (59–72 yr) men	CD34^+^VEGFR2^+^ cells (FC) EPC migration	Reduced number and migration of EPCs in sedentary old versus sedentary and endurance trained young, no difference in endurance trained old versus young men

FC: flow cytometry, CFU: colony forming units.
